# Lived experiences of cardiovascular postoperative care in pediatric and neonatal critical care: The parents’ experience

**DOI:** 10.15649/cuidarte.5175

**Published:** 2026-08-14

**Authors:** Sergio Andres Acuña-Caicedo, Geraldine Tatiana Piraquive-Niño, Leidy Paola Triana-Monroy

**Affiliations:** 1 Universidad El Bosque, Bogotá, Colombia. E-mail: saacuna@unbosque.edu.co Universidad El Bosque Bogotá Colombia saacuna@unbosque.edu.co; 2 Fundación Universitaria Sanitas, Bogotá, Colombia. E-mail: gtpiraquiven@unal.edu.co Fundación Universitaria Sanitas Bogotá Colombia gtpiraquiven@unal.edu.co; 3 Hospital Universitario San Ignacio, Bogotá, Colombia. E-mail: trianam-lpaola@javeriana.edu.co Hospital Universitario San Ignacio Bogotá Colombia trianam-lpaola@javeriana.edu.co

**Keywords:** Parents, Child Hospitalized, Life Experiences, Thoracic Surgery, Intensive Care Units, Pediatric, Padres, Niño Hospitalizado, Experiencias de Vida, Cirugía Torácica, Unidades de Cuidado Intensivo Pediátrico, Pais, Criança Hospitalizada, Experiência de Vida, Cirurgia Torácica, Unidades de Terapia Intensiva Pediátrica

## Abstract

**Introduction:**

Heart disease in childhood is the fourth leading cause of illness and, in many cases, requires complex surgical procedures and intensive care unit care. This situation has a significant impact on family caregivers, especially at the psychosocial and emotional levels, due to the uncertainty about the children's prognosis. Therefore, it is essential to provide support to facilitate coping with this critical experience.

**Objective:**

To describe the experiences of parents and family caregivers caring for children postoperatively after cardiovascular surgery, hospitalized in in pediatric intensive care units and neonatal intensive care units of a fourth-level institution, during 2023.

**Materials and Methods:**

A qualitative study with a descriptive phenomenological approach based on the Colaizzi method. In-depth interviews were conducted with family caregivers, selected by convenience, until data saturation was achieved. Data collection was conducted by the researcher and data analysis was supported by Nvivo software. The information was structured into core themes and subthemes through methodological triangulation.

**Results:**

Ten mothers, aged 23 to 46 years, mostly from stratum 2, were interviewed. The children ranged in age from 14 days to 6 years, with common surgical diagnoses such as closure of patent ductus arteriosus.

**Discussion:**

The analysis yielded six categories, 15 codes, and common themes such as the need for effective communication and the coping strategies employed.

**Conclusion:**

Caregivers require professionals with assertive communication skills who build trust, include them in care, and respect their beliefs to reduce the emotional impact.

## Resumen

**Introducción:**

Las cardiopatías en la infancia constituyen la cuarta causa de enfermedad y, en muchos casos, requieren procedimientos quirúrgicos complejos y atención en unidades de cuidado intensivo. Esta situación genera un impacto significativo en los cuidadores familiares, especialmente a nivel psicosocial y emocional, debido a la incertidumbre sobre el pronóstico de los niños. Por ello, se hace fundamental brindar apoyo que facilite el afrontamiento de esta experiencia crítica.

**Objetivo:**

Describir las vivencias de los padres y cuidadores familiares frente al cuidado de niños en postoperatorio de cirugía cardiovascular, hospitalizados en unidades de cuidado intensivo pediátrico y neonatal de una institución de cuarto nivel, durante el año 2023.

**Materiales y métodos:**

Estudio cualitativo de enfoque fenomenológico descriptivo, basado en el método de Colaizzi. Se realizaron entrevistas a profundidad a cuidadores familiares, seleccionados por conveniencia hasta la saturación de la información. La recolección fue realizada por el investigador y el análisis de datos se apoyó en el software Nvivo. Se estructuró la información en temas centrales y subtemas mediante triangulación metodológica.

**Resultados:**

Se entrevistaron 10 madres, de entre 23 y 46 años, en su mayoría del estrato 2. Los niños tenían entre 14 días y 6 años, con diagnósticos quirúrgicos frecuentes como cierre de ductus arterioso persistente.

**Discusión:**

El análisis arrojó seis categorías, 15 códigos y temas comunes como la necesidad de comunicación efectiva y las estrategias de afrontamiento empleadas.

**Conclusiones:**

Los cuidadores requieren profesionales con habilidades comunicativas asertivas, que les generen confianza, los incluyan en el cuidado y respeten sus creencias para reducir el impacto emocional.

## Resumo

**Introdução:**

A cardiopatia congênita é a quarta principal causa de morbidade na infância e, em muitos casos, requer procedimentos cirúrgicos complexos e cuidados intensivos. Essa situação impacta significativamente os familiares cuidadores, principalmente nos âmbitos psicossocial e emocional, devido à incerteza quanto ao prognóstico da criança. Portanto, é essencial oferecer suporte que facilite o enfrentamento dessa experiência crítica.

**Objetivo:**

Descrever as experiências de pais e familiares cuidadores de crianças no período pós-operatório de cirurgia cardiovascular, internadas nas Unidades de Terapia Intensiva Pediátrica e Neonatal de uma instituição terciária de saúde durante o ano de 2023.

**Materiais e métodos:**

Estudo qualitativo com abordagem fenomenológica descritiva, baseado no método de Colaizzi. Foram realizadas entrevistas em profundidade com familiares cuidadores, selecionados por amostragem de conveniência, até a saturação dos dados. A coleta de dados foi realizada pela pesquisadora e a análise dos dados foi feita utilizando o software NVivo. As informações foram estruturadas em temas centrais e subtemas utilizando triangulação metodológica.

**Resultados:**

Dez mães, com idades entre 23 e 46 anos, em sua maioria do estrato socioeconômico 2, foram entrevistadas. As crianças tinham idades entre 14 dias e 6 anos, com diagnósticos cirúrgicos frequentes, como oclusão de canal arterial patente.

**Discussão:**

A análise resultou em seis categorias, 15 códigos e temas comuns, como a necessidade de comunicação eficaz e as estratégias de enfrentamento empregadas.

**Conclusões:**

Os cuidadores necessitam de profissionais com habilidades de comunicação assertiva que inspirem confiança, os incluam no processo de cuidado e respeitem suas crenças para reduzir o impacto emocional.

## Introduction

Many diseases affecting the pediatric population are managed in intensive care units (ICUs), where continuous hemodynamic monitoring is conducted due to life-threatening clinical instability. For such cases, various invasive and non-invasive interventions are used, such as mechanical ventilation, sedation, muscle relaxants, central catheters, as well as feeding tubes and urinary drainage catheters^1^.

This practice has a strong emotional impact on parents and family caregivers, who experience sadness, anxiety, frustration, and uncertainty about the health of their children^1^,^2^. The mere knowledge that their child will be admitted to an ICU might induce fear regarding the prognosis and outcome of the disease. Therefore, it is essential to provide emotional support from the time of admission in order to help caregivers cope with the situation.

One of the most frequent conditions in pediatrics is congenital heart defects. These diseases are the fourth leading cause of childhood morbidity and, in many cases, require complex surgical interventions with recovery in the ICU^3^-^5^. ICU stays allow the monitoring of possible postoperative complications, restoration of cardiac function, and fulfillment of the child’s physiological needs. This level of care requires the intervention of a trained interdisciplinary team, wherein nurses, being the staff with the greatest contact with the critically ill child, play an essential role in the direct and comprehensive care of the patient and family^6^.

In view of this reality, it is essential to learn about the lived experiences of primary caregivers of children undergoing cardiovascular surgery, in order to delve deeper into their care-related experiences. This knowledge can enrich nursing practice from a reflective, ethical, and humanized perspective within a qualitative research approach. This methodological stance contributes to disciplinary advancement by allowing the phenomenon to be addressed through the analysis of the relationships and meanings constructed around critical care^7^.

Likewise, it is understood that the caregiver-patient relationship is disrupted and reconfigured during the ICU stay. This situation can be analyzed through Merle Mishel’s theory of uncertainty in illness, which proposes that caregivers go through coping or buffering processes in the face of uncertain events. In this context, interventions that improve their experience and promote emotional and relational well-being during the child’s treatment can be implemented^8^.

The objective was to describe the experience of parents regarding postoperative cardiovascular care in pediatric critical care at a fourth-level institution during the second and third quarters of 2023.

## Materials and Methods

This study corresponds to descriptive qualitative research conducted under a phenomenological approach. Its purpose was to gain an in-depth understanding of the experience of parents of children hospitalized in pediatric intensive care units (PICU) and neonatal intensive care units (NICU) after cardiovascular surgery. This methodological perspective makes it possible to explore the meanings attributed by caregivers to their experience, recognizing the subjectivity and complexity of the phenomenon under study^9^.

**Participants and sampling**

Participants were selected by convenience sampling; only those who presented the phenomenon of interest were included in the study. Theoretical saturation was used as a criterion to determine the number of participants, i.e., the collection process was continued until no new categories or relevant ideas emerged^9^.

Inclusion criteria were as follows: being a parent or primary family caregiver of a child hospitalized in PICU or NICU after cardiovascular surgery and being of legal age. Caregivers with cognitive or communication impairments that prevented an adequate interview were excluded.

**Data collection techniques and instruments**

Data were collected through semi-structured interviews, recorded with prior consent, together with nonverbal observations documented in a field journal. One researcher conducted the interviews, while a second observer noted participants’ gestures, attitudes and emotional reactions, aspects that are fundamental to the phenomenological approach^9^,^10^.

1. Planning: Identification of potential participants from PICU and NICU admission records, followed by telephone calls to explain the objectives of the study and arrange interviews.

2. Opening: Signing of the informed consent form, completion of the sociodemographic form, and explanation of the researchers’ role.

3. Development: Conduct of the interview (20 to 60 min), ensuring an environment of respect, active listening, and emotional support.

4. Closing: A space for gratitude, validation of contributions, and general feedback on the process.

**Data analysis**

The analysis followed rigor criteria for qualitative research, ensuring credibility, transferability, confirmability, and adequacy^9^,^10^. Colaizzi’s phenomenological method was used, which included exhaustive reading of the transcripts, extraction of significant phrases, formulation of meanings, organization into categories, and elaboration of an exhaustive description of the experience, later validated with some participants. Interviews were transcribed verbatim, and repeated readings were conducted by researchers. This approach made it possible to identify the first units of meaning. Coding and categorization were performed using the NVivo 12 Pro software, and information was organized based on the emerging codes. Word clouds were constructed to identify the most frequent terms, facilitating initial categorization. In total, six main categories and 15 codes were defined, derived from approximately 55 significant phrases extracted from the interviews. The complete dataset is available for open access and consultation in Mendeley Data^11^.

**Ethical considerations**

This study was classified as minimal risk according to Resolution 8430 of 1993 of the Colombian Ministry of Health^12^. However, given that emotionally sensitive experiences were addressed, a care protocol was established in the event of emotional distress during the interview, with support from the institution’s psychology, spirituality, and social work services.

The study was approved by the Ethics Committee of the Faculty of Medicine of Pontificia Universidad Javeriana, in conjunction with Hospital Universitario San Ignacio, under code 2023/158, minutes 13/2023.

## Results

**Participants’ sociodemographic characteristics**

Ten individuals participated in the study. Ages ranged from 23 to 46 years, with most participants in the 21 to 25 and 31 to 35 age groups. In terms of socioeconomic level, five of the participants belonged to stratum 2, three to stratum 1, and two to strata 3 and 4. In terms of education, two participants had completed primary education, five had completed high school, two had an undergraduate degree, and one had a postgraduate degree.

**Clinical characteristics of children**

Among the children, 20% were neonates (1 to 30 days old), 60% were between 1 month and 2 years old, and the remaining 20% were between 5 and 11 years old. The most frequent surgical interventions were closure of patent ductus arteriosus (PDA), pulmonary artery banding, and correction of coarctation of the aorta. Postoperative PICU stay ranged from 4 to 40 days, depending on the type of surgery and the occurrence of clinical complications.

**Categorization and thematic analysis**

After verbatim transcription of the interviews and their analysis using the NVivo version 12 Pro software, a word cloud was constructed to identify the most relevant terms associated with caregivers’experience. From this analysis, six main categories and fifteen codes were established, emerging from about 55 significant phrases. The following categories were identified: emotions and feelings, communication, stressors, participation in care, role and relationships, and coping^7^.

**Figure 1:**
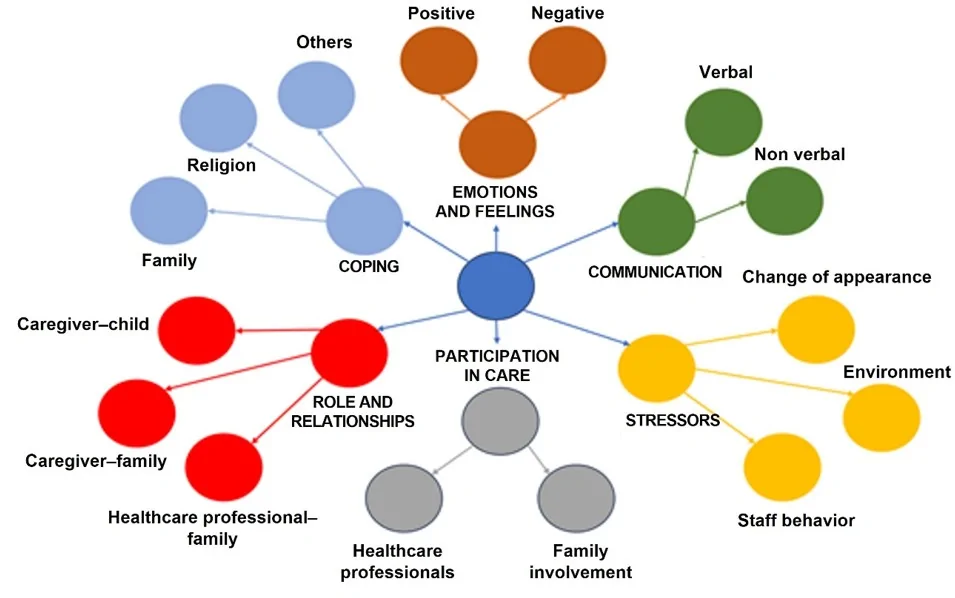
Cluster diagram of category emergence

**Category 1: Emotions and feelings**

Based on the opening question: “How did you feel when you saw your child in the intensive care unit for the first time?”, the initial emotional impact on caregivers was explored. Two codes emerged:

**Negative emotions and feelings: ** Caregivers expressed distress, anxiety, and sadness in response to their child’s hospitalization, evidencing the strong emotional impact of the experience. Testimonials:

*“I felt so distressed, anxious, concerned. It is not easy to have a child in... an intensive care unit,” E6P6N4.*

*“I felt really sad,” E7P7P3. “...Fear... I cried,” E8P8P4.*

**Positive emotions and feelings:** Caregivers also highlighted feelings of relief, joy, and reassurance in response to the child’s clinical progress and the care received. Examples:

*“... She was extubated, so for me that was a relief...,” E3P3P2. “... A lot of joy and a sense of gratitude,” E5P5N3. “What I liked most was doctors’ human warmth, which is something you don’t see anywhere else...,” E3P3P2. “... A lot of tranquility” E7P7P3.*

**Category 2: Communication**

Communication was identified as a key aspect for adaptation to the PICU environment. Two types of communication were identified:

**Verbal communication:** Participants highlighted the clarity and continuity of the information provided by the healthcare team, which favored trust and understanding of the process. Testimonials:

*“They always explained what they were going to do and how,” E3P3P2. “The specialists, the intensivists and the head nurses always explained to us the procedure, how invasive it would be, its risks, the approximate number of machines he would be connected to afterward” E9P9P5. “They explained it to me very well,” E7P7P3.*

**Non-verbal communication:** The caregivers identified gestures of support and closeness that conveyed trust, such as “Seeing that the head nurses and the medical team would sometimes celebrate with us with the same joy,” E9P9P5. However, when analyzing the relationship between staff and participants, information flow showed some weaknesses. In this sense, actions by some team members stood out, and their abrupt manners somewhat affected participants. The following phrases were recorded:

*“Because I knew that they were going to respond harshly, and I didn’t want to upset them, or myself...,” E6P6N4. “Because you feel like you got it wrong by asking, or that you should not have done it, or that you’d better ask” E6P6N4. “As if they were saying ‘don’t ask me, ask this or that person, but not me!’,” E6P6N4.*

**Category 3: Stressors**

This category grouped items that increased emotional distress during hospitalization:

**Change in the child’s appearance:** Caregivers expressed the emotional impact of watching their children’s physical appearance change during hospitalization. They made comments such as:

*“...intubated, I mean, seeing him swollen was very painful for me...,” E2P2P1. “...He had many things around, he had so many things on him, like the catheter, and that always made you feel afraid,” E3P3P2. “...So many machines, so many beeps, seeing him intubated, it was very, very hard...,” E9P9P5.*

**Hospital environment:** Caregivers’ accounts reveal both positive aspects and limitations of the hospital environment. For some, the infrastructure offered a sense of security: “And to expand the unit ... partly because you feel a little more protected,” E1P1N1. However, shortcomings associated with human resources and accommodation conditions were pointed out:

*“...I feel that sometimes more staff is needed. I mean sometimes, I see that their workload is much heavier than it should normally be...,” E3P3P2. “In the unit where I was, I had to sleep sitting down, next to her...,” E4P4N2.*

**Professional behavior:** Participants highlighted differences in the way health personnel treated them, ranging from empathy to a lack of sensitivity. Some pointed out the absence of a human supportive approach in communication:

*“[Doctors]... sometimes didn’t seem to have the heart to tell me, this is what is going to happen, this, this, and this…,” E4P4N2. “...They don’t address you with kindness, like, with an understanding of the situation, like with empathy,” E6P6N4. However, the positive attitude of some professionals was also acknowledged: “...Some shifts have people who are always friendly and some others don’t,” E6P6N4.*

**Category 4: Participation in care**

Caregivers positively valued the opportunities for physical and emotional contact with their children during the PICU stay, which strengthened their parent-child bond and allowed them to take an active role in recovery.

**Healthcare professionals:** Caregivers highlighted the commitment and dedication of the healthcare team, especially the nurses, whose constant presence provided trust and security. The following phrases were recorded:

*“...The nurses were very attentive to my daughter, and what I liked was that I arrived the next day and everyone knew about my child’s situation...,” E1P1N1. “...They were keeping a close eye on my baby every day, checking whether her blood pressure was dropping, her saturation, they were always there watching her,” E2P2P1. “They were very attentive to him, they’ve saved his life here many times,” E5P5N3.*

**Family member involvement:** Caregivers positively valued the opportunities to participate in care and the information shared by the healthcare team. They uttered phrases such as:

*“...She taught me how to put her to sleep...,” E3P3P2. “Because, yes, obviously we are all a team,” E6P6N4. “...I think it’s very important that they tell me,” E7P7P3. “...The head nurses, especially when it came to medications and keeping track of all those things, I think their care was appropriate, all the nurses who cared for my daughter would explain to me: I’m going to administer this medication, we’re going to give her this medication; this is for this and this, and they prescribed this many doses; I thought that was great,” E1P1N1.*

**Category 5: Roles and relationships**

During hospitalization, three main types of human relationships were identified that significantly impacted caregivers’ experience:

**Healthcare professional-caregiver relationship:** The caregivers highlighted the support received from the nurses and other professionals, whom they perceived as close and constant companions:

*“...The head nurses, the nursing assistants (...) are a great support,” E1P1N1. “...They were always there for me; they were angels...,” E2P2P1. “The nursing team, the intensivists, the pediatricians, were also the ones who lifted our spirits,” E9P9P5. “I will never get tired of saying that San Ignacio Hospital was also like a family for my home, for my husband, and for my son,” E9P9P5. Among the differences within the team, parents reported: “...I felt closer... to the head nurse and to the nursing assistants...,” E3P3P2. “...Because with the doctor, I don’t know, during rounds, his work was like... he came, examined, asked you questions, but you don’t develop that same level of trust,” E3P3P2. “I perhaps received more attention and support from a therapist (respiratory therapist) than from a nursing assistant and a doctor,” E6P6N4.*

**Caregiver-family relationship:** Testimonials evidenced the tensions arising when caregivers had to balance care for the hospitalized child and their family responsibilities. Some reported difficulties in visiting their child in the ICU:

*“I would say to my mother, ‘I really cannot go in and see her there; it is very hard for me’,” E4P4N2. “I have a daughter, and I had to leave her, not alone because she was with my sisters, but it wasn’t the same...,” E8P8P4. “Well, we took turns with my husband, but he had to travel, so it was my turn.” E8P8P4. “...to leave our other 3 children...,” E9P9P5.*

**Parent-child relationship:** Caregivers expressed deep emotional connection with their children, marked by fear, protection, and a sense of purpose in the experience. Expressions such as the following were recorded:

*“My God, may she not be in pain,” E1P1N1. “I think that was... like a purpose I have with her in life,” E4P4N2. “At first I thought it was something very risky and that I was going to lose my child,” E5P5N3.*

**Category 6: Coping**

The coping process was structured around various strategies that enabled caregivers to cope with the emotional impact of hospitalization:

**Familysupport:** Caregivers highlighted the importance of the support network during hospitalization, both for the child and the family. They uttered phrases such as:

*“The child never really loses that sense of familiarity,” E3P2P2. “Well, we have a functional home with my husband and we were here, taking turns 24 hours a day,” E9P9P5. “... I really would have liked to be with my mother. Yes, but she’s with my other child...,” E6P6N4. “... The care... is very good; however, a support network of parents would be nice, even though there are psychology meetings on Wednesdays,” E6P6N4.*

**Religion and spirituality:** Faith became a fundamental coping resource for the caregivers, who attributed recovery and strength throughout the process to God. The following testimonials were recorded:

*“Thanks to God, my son came out of the surgery very well and my Lord Jesus worked wonderfully,” E6P6N4. “Thanks to God everything went very well,” E3P3P2. “Because I fully relied on God,” E6P6N4.*

**Other strategies:** Some caregivers shared personal and external resources that helped them cope with the experience. One of them was early preparation:

*“I had to prepare very well, because as soon as I had her, she was going into intensive care,” E4P4N2. “Suddenly hearing those words, many times alleviated the way us parents handled our emotions,” E6P6N4.*

## Discussion

When analyzing emotions and feelings, negative emotions and feelings stood out: Participants expressed distress, fear, despair, sadness, anxiety, and pain. The literature describes this experience as an “emotional roller coaster”, with divergent thoughts and changing feelings, especially in the face of the child’s critical condition^13^-^18^. Positive emotions and feelings were also identified: feelings of relief, confidence, and satisfaction related to the success of the surgery and the care provided by healthcare staff. Although these feelings are less frequently reported in the literature, some studies highlight that the quality of care received generates peace of mind in caregivers^13^-^19^.

Communication was identified as a key aspect for adaptation to the PICU environment. Two types of communication were identified. The first was verbal communication, wherein caregivers valued clear, anticipatory information about the surgical procedure, the child’s appearance, the devices used, and the clinical condition, which allowed them to prepare emotionally for the postoperative reunion.

Literature confirms the importance of effective communication as a coping strategy in contexts of high uncertainty^13^-^18^. The second was non-verbal communication: staff attitudes and expressions also influenced the perception of care. Although there is scarce scientific literature on this topic^20^, lcaregivers noted that gestures such as a smile, eye contact, or a calm tone were comforting and facilitated interaction.

Stressors grouped the elements that increased emotional distress during hospitalization, including changes in the child’s appearance. In this sense, the presence of invasive devices generated feelings of pain, sadness, and helplessness. Previous studies report that these changes in body appearance intensify perception of the child’s vulnerability^15^,^18^. The hospital environment was also identified as a stressor: caregivers described equipment noise, intense lighting, confined space, and constant staff activity as stressors. Literature suggests that these aspects contribute to an overwhelming experience and hinder patients’ and caregivers’ rest^14^-^19^,^21^,^22^. Healthcare professionals’ behavior was another stressor, and in this regard, some caregivers reported cold or unempathetic treatment by certain professionals, which increased their level of stress. This perception is related to information inconsistencies, insensitive attitudes, and visible stress by the staff during critical patient care^16^-^19^,^21^,^23^,^24^.

Caregivers positively valued the opportunities for physical and emotional contact with their children during the PICU stay, which strengthened the parent-child bond and allowed them to play an active role in recovery. In addition, caregivers highlighted the humanized care provided by doctors, nurses, assistants, and therapists, who facilitated safe closeness to the child. The perception of competent professional care generated trust in the care provided^19^,^22^,^25^-^28^. Involvement of family members, reflected in the possibility of participating in daily activities such as bathing or feeding, was described as important. This participation reduced their fear of causing harm and promoted the perception of control over the situation. Literature highlights the role of nursing in facilitating this interaction, promoting active, safe, and emotionally restorative participation by caregivers^13^,^16^-^21^.

During hospitalization, three main types of human relationships were identified that significantly impacted the caregiver experience. The first type was the relationship between healthcare professionals and caregivers, which proved to be a fundamental source of support. Communication quality, empathy, and respect were key to establishing a relationship of trust. However, some caregivers reported negative experiences with certain staff members, which affected their overall perception of care. The literature suggests that empathic interaction between healthcare staff and caregivers is essential for a satisfactory experience, facilitating understanding of the process and emotional adaptation^16^,^21^,^22^. The second type was the relationship between caregivers and families. Support from family members was recognized as vital for the emotional, economic, and logistical sustenance of the primary caregiver. However, prolonged hospitalization also generated tensions, because some parents had to leave their other children or experienced responsibility overload. Previous studies show that family separation can generate feelings of guilt, fatigue, and isolation, affecting the emotional well-being of the caregiver^13^,^15^-^18^,^23^,^24^. The third type of relationship was the parent-child relationship. Physical contact, expressions of affection, and participation in direct care were described as strategies to alleviate parental stress. However, some caregivers expressed fear or anxiety about their child’s critical condition, which generated emotional ambivalence. The literature also reports this tension between the desire to protect the child and the fear of intensifying their suffering, as well as admiration for the child’s strength in the face of surgery^13^,^18^,^24^.

The coping process was structured around various strategies that allowed caregivers to manage the emotional impact of hospitalization. Family support played a significant role, as the companionship and practical help of relatives allowed caregivers to share the emotional burden, receive comfort, and strengthen their resilience. Literature supports the importance of these networks as facilitators of coping and adaptation to complex situations^13^,^18^. One of the coping approaches involved religion and spirituality. Faith was expressed as a source of meaning and hope, helping to re-signify the experience as a “test” or “divine will.” This perspective made it possible to alleviate suffering, accept uncertainty, and value the role of healthcare staff as an instrument of divine intervention. Several studies agree that spirituality plays a protective role against emotional distress in contexts of high uncertainty^28^. Other coping mechanisms included the use of humor, positive thinking, seeking shared experiences with other caregivers, and reinterpreting the event as an opportunity for growth. These strategies are supported by the literature as effective mechanisms to mitigate the psychological impact of the intensive care environment^18^.

## Conclusions

The experience of parents and family caregivers of children in the postoperative period following cardiovascular surgery in pediatric intensive care units is characterized as emotionally intense, uncertain, and challenging. From admission, caregivers deal with a reality that transforms their daily lives, marked by fear of loss, uncertainty regarding the prognosis, and adaptation to a highly technical environment specialized in pediatric cardiovascular care.

During the process, ambivalent emotions emerge. Initially, fear, sadness, and distress predominate, which progressively turn into hope and tranquility with the clinical evolution of the child and the bond built with the healthcare team. This emotional transition highlights the importance of coping strategies and professional support to facilitate adaptation.

Communication is recognized as a central element of the experience. When it is clear, empathetic, and respectful, whether verbal or nonverbal, it helps reduce anxiety and encourages the active participation of caregivers in the child’s care.

Interpersonal relationships with the healthcare team, the hospitalized child, and other family members play a key role. Maintaining a close bond through inclusion in pediatric cardiovascular care is experienced as an emotionally accurate and protective mechanism in the face of suffering.

Finally, spiritual and family support emerges as a resource for resilience, providing meaning and strength in the midst of adversity. These findings demonstrate the need for a holistic and comprehensive approach in pediatric cardiovascular care, wherein nurses and the healthcare team recognize caregivers’ suffering, promote their active involvement, and strengthen a family-centered model of care.
